# Neural correlates of altered feedback learning in women recovered from anorexia nervosa

**DOI:** 10.1038/s41598-017-04761-y

**Published:** 2017-07-14

**Authors:** Franziska Ritschel, Daniel Geisler, Joseph A. King, Fabio Bernardoni, Maria Seidel, Ilka Boehm, Richard Vettermann, Ronald Biemann, Veit Roessner, Michael N. Smolka, Stefan Ehrlich

**Affiliations:** 1 Division of Psychological and Social Medicine and Developmental Neuroscience, Faculty of Medicine, Technische Universität Dresden, Dresden, Germany; 2 Eating Disorder Treatment and Research Center, Department of Child and Adolescent Psychiatry, Faculty of Medicine, Technische Universität Dresden, Dresden, Germany; 3 Institute of Clinical Chemistry and Pathobiochemistry, Otto-von-Guericke University, Magdeburg, Germany; 4 Department of Psychiatry and Neuroimaging, Technische Universität Dresden, Dresden, Germany

## Abstract

Anorexia nervosa (AN) is associated with exaggerated self-control and altered reward-based decision making, but the underlying neural mechanisms are poorly understood. Consistent with the notion of excessive cognitive control, we recently found increased dorsal anterior cingulate cortex (dACC) activation in acutely ill patients (acAN) on lose-shift trials in a probabilistic reversal learning (PRL) task. However, undernutrition may modulate brain function. In attempt to disentangle trait from state factors, the current fMRI study investigated cognitive control in recovered patients (recAN). Thirty-one recAN and 31 healthy controls (HC) completed a PRL task during fMRI. Based on previous findings, we focused on hemodynamic responses during lose-shift behaviour and conducted supplementary functional connectivity analysis. RecAN showed elevated lose-shift behaviour relative to HC. On the neural level, recAN showed normal dACC responses, but increased activation in fronto-parietal control regions. A trend for increased coupling between frontal and parietal regions of interest was also evident in recAN. The current findings in recAN differ from those in our previous study in acAN. While aberrant dACC response to negative feedback may be a correlate of the underweight state in acAN, impaired behavioural adaptation and elevated activation of cognitive control regions in recAN is suggestive of altered neural efficiency.

## Introduction

Anorexia nervosa (AN) is a serious eating disorder characterized by an intense fear of weight gain, body image distortion and severe weight loss, typically by restrictive eating behaviour. Individuals with AN are often described as perfectionistic and having a harm avoidant personality style^1^ even after recovery^2^. These cognitive styles and behavioural schemata may have adverse effects on AN patients’ decision making capacity, e.g. they may interfere with the ability to learn from experience^3, 4^. A growing body of research on decision making and cognitive control in AN has reported impairments in reward-related decision making^5^ and altered engagement of executive control brain circuitry in acutely ill patients^6–8^. In individuals recovered from AN (recAN), however, studies on reward-related decision making are still relatively scarce. Some behavioural investigations suggest persistent impairments in recAN^9–11^, while others found normalized decision making in fully remitted AN patients^12^. Recent reviews concluded that in large part the data are mixed^5, 13^. The few available fMRI studies in recAN seem suggestive of persisting disturbances in brain regions associated with cognitive control. For example, resting state studies^14–16^ and a task-based study on simple reward processing^17^ showed alteration in cognitive control regions in recAN.

For a deeper understanding, we studied probabilistic reversal learning (PRL) - which probes characteristics pertinent to AN including (reward-based) decision making, cognitive control, and flexibility of behaviour – with functional magnetic resonance imaging (fMRI). Reversal learning mimics the ability to survive in a dynamically changing environment and requires ongoing evaluation of action outcomes^18^. In PRL paradigms, participants learn to respond to a stimulus to receive a reward, but they have to detect implicit changes in stimulus-reward contingencies and react to the alternative stimulus when contingencies are reversed. A probabilistic feedback schedule mirrors real-life as it simulates the uncertainty in the natural environment, which may be challenging for AN patients.

Using such a task, we previously found evidence for increased neural responses in dorsal anterior cingulate cortex (dACC) in acutely ill AN patients (acAN)^19^. In particular, acAN activated the dACC more than healthy controls during confrontation with negative feedback that incurred a change in behaviour (lose-shift behaviour), while general task performance was comparable between groups. We interpreted these findings as suggestive of increased cognitive control, in particular, increased monitoring for the need to adjust performance strategies.

However, severe undernutrition in AN has been shown to be associated with (pseudo-) atrophic changes in grey matter^20, 21^, which may also affect performance on cognitive tasks^22^ and brain function. Further, a large number of rather drastic changes in the endocrine system (e.g. decreased secretion of gonadal hormones, suppressed leptin levels) have been associated with the underweight state^23, 24^. Altered levels of hormones and neurotransmitters in the acute AN phase were found to influence psychiatric symptoms^25–29^. Thus, it is possible that the detected effects in acAN in our previous study were merely due to acute undernutrition. Therefore, given the importance of distinguishing between state and trait factors in the study of AN^30^, the aim of the current study was to investigate, if altered activation in cognitive control regions in particular the dACC persist in patients recovered from AN. Following our previous findings^19^, we focused on activation associated with negative feedback calling for behavioural adjustment.

## Results

### Demographics

Demographic characteristics and comparisons of behavioural measures are summarized in Table 1. As expected, recAN and healthy control women (HC) did not differ in age, BMI-SDS and leptin levels. Further, no group differences were found in IQ, but recAN participants showed some residual eating disorder symptoms (EDI-2).

**Table 1:** Descriptive statistics. Demographic, clinical, and endocrine parameters (results of independent paired T-test; p < 0.05).

	recAN	HC	T	p
**Demographic variables**				
N	31	31	—	—
Age	22.31 ± 2.8	22.05 ± 3.0	−0.354	0.725
IQ	109.35 ± 9.3	110.73 ± 8.0	0.619	0.538
**Clinical variables**				
BMI	20.99 ± 1.9	21.30 ± 2.1	—	—
BMI-SDS	−0.452 ± 0.62	−0.332 ± 0.63	0.780	0.439
Minimal lifetime BMI	14.30 ± 1.8	20.22 ± 2.1	11.731	0.000
Depression score (SCL-90R)	0.53 ± 0.6	0.35 ± 0.6	−1.174	0.245
EDI-2 - perfectionism	2.94 ± 0.9	2.62 ± 1.0	−1.302	0.198
EDI-2 - total score	20.25 ± 5.7	16.69 ± 3.3	−2.978	0.005
Leptin [ng/ml]	10.17 ± 6.1	10.41 ± 7.2	0.136	0.893

BMI-SDS was used for statistical analysis instead of BMI because the former provides an index of weight to height ratio that is corrected for age and gender^100, 101^. Abbreviations: recAN = recovered anorexia nervosa patients; HC = healthy controls; IQ = intelligence quotient; BMI-SDS = body mass index standard deviation score; EDI-2 = Eating Disorder Inventory. Displayed are means ± standard deviations.

### Behavioural Data

Performance data are summarized in Table 2. HC showed a higher overall hit ratio, higher number of contingency reversals and therefore a higher total win. RecAN showed a higher rate of lose-shift behaviour and a statistical trend for a higher rate of win-shift behaviour. There was no significant group difference in persistence. Correlational analysis did not reveal any significant associations between task performance and clinical variables. For complete details see Supplementary Information (SI) 2.1.

**Table 2:** Task related variables (results of independent paired T-test; p < 0.05).

	recAN	HC	T	p
Lose-shift	28.26 ± 7.0	23.61 ± 7.7	−2.487	0.016
Win-shift	5.65 ± 6.2	3.16 ± 3.6	−1.933	0.059
Hit ratio	0.680 ± 0.06	0.711 ± 0.05	2.236	0.029
Contingency reversal	8.35 ± 2.1	9.52 ± 1.9	2.292	0.025
Total win [€]	4.68 ± 2.7	6.06 ± 2.3	2.163	0.035
Persistence	2.246 ± 0.9	2.405 ± 0.8	0.700	0.487

Abbreviations: recAN = recovered anorexia nervosa patients; HC = healthy controls; lose-shift = negative feedback incurring a change in behaviour; win-shift = positive feedback incurring a change in behaviour. Displayed are means ± standard deviations. For more details on additional task performance measures see SI Table S1.

### Imaging Data – general linear model

In accordance with Hampton *et al*.^31^, exploratory analysis of main effect of the lose-shift condition revealed significant activation in brain regions typically associated with processing of negative feedback including anterior cingulate cortex, insula, and dorsolateral prefrontal cortex (SI 2.2).

**Figure 1:**
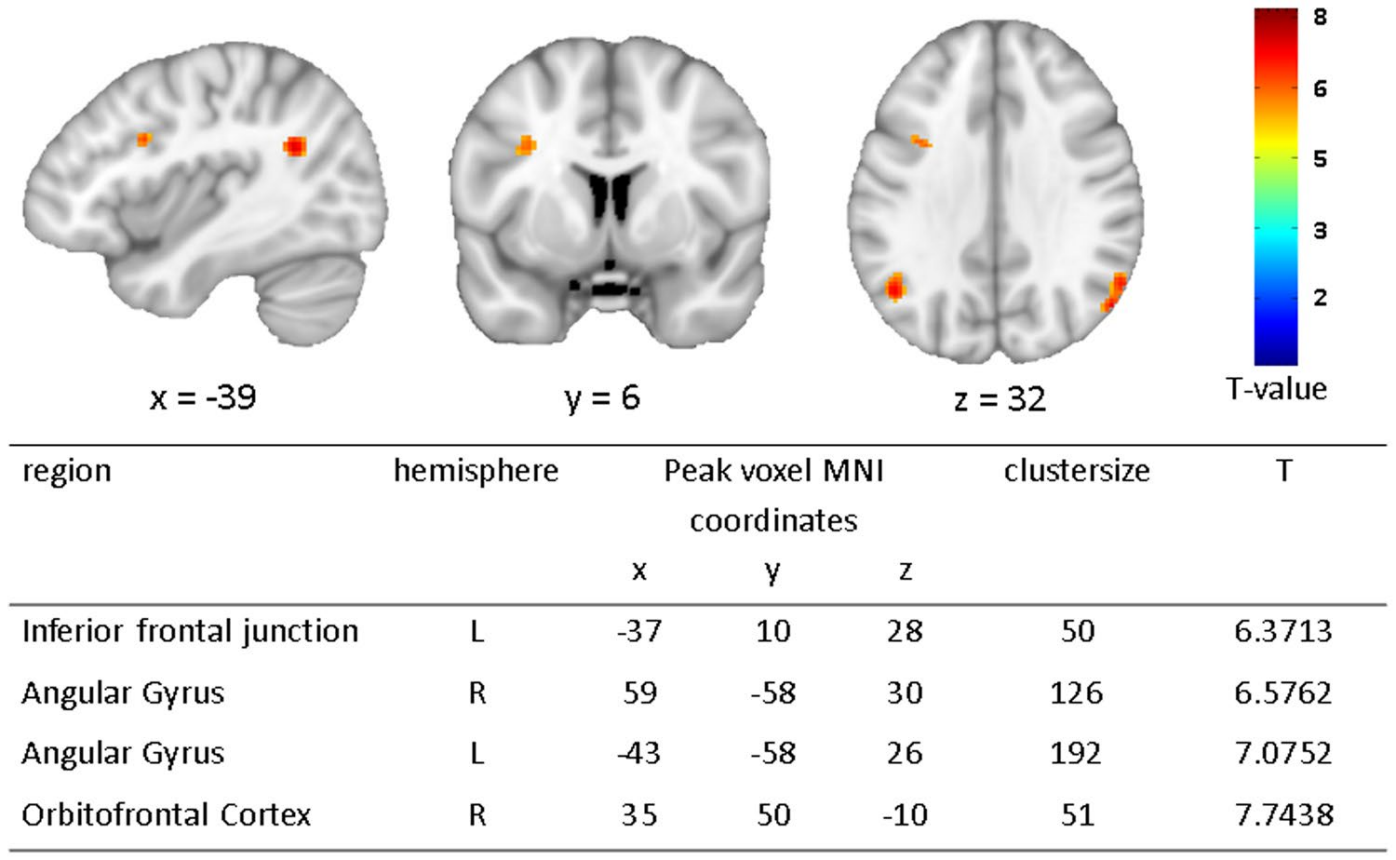
fMRI results showing group differences (recAN > HC) during lose-shift behaviour (FWE; α = 0.01; k = 50voxels). In the upper panel brain maps are shown and in the lower panel local peaks of clusters are listed that are more activated during lose-shift in recAN. Abbreviations: lose-shift = negative feedback incurring a change in behaviour; L = left; R = right; HC = healthy control; recAN = recovered anorexia nervosa patient.

Group comparison revealed no group difference in dACC activation as previously reported in acAN^19^. However, activation in the bilateral angular gyrus (AG), left inferior frontal junction (IFJ), and right orbitofrontal cortex (OFC; Fig. 1) was elevated in recAN. Additional analysis (group comparisons), reported in SI 2.3, excluding one patient who had a history of OCD or covarying for comorbid depressive symptoms confirmed the original results. Analyses of correlation between activation in the identified clusters (regions) and clinical measures (see Method section for details) did not yield any significant associations (all r < 0.53; n.s.).

Given our initial hypothesis regarding the dACC based on the findings in acAN of Geisler *et al*.^19^ (see Method section) and a lack of group differences on whole brain level, we conducted an exploratory analysis. To this end, we extracted parameter estimates from this region of interest (ROI) to further scrutinize possible activation patterns in recAN. Specifically, we explored whether group differences in dACC activation emerge when taking the behavioural differences in performance (represented by hit ratio) into account in an ANCOVA. Indeed, a group × hit ratio interaction [F(2,59) = 10.91, p = 0.002] characterized by a positive correlation between hit ratio and dACC response in recAN and the opposite pattern in HC was revealed (SI 2.5, Figure S5).

### Connectivity analysis

**Figure 2:**
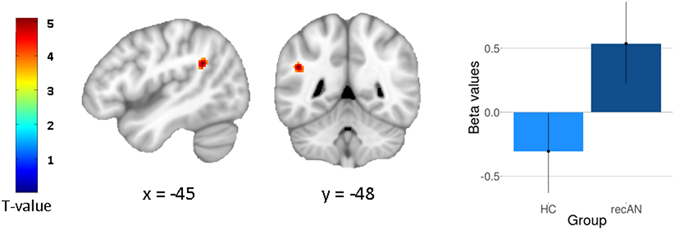
gPPI results. Statistical maps (whole-brain analysis, p < 0.001 uncorrected, cluster extent k ≥ 30voxels) showing regions of group differences in functional connectivity for the lose-shift condition (seed region: IFJ) and extracted beta values from AG cluster. Global peak: x = −45, y = −48, z = 28 [T(2,120) = 4.4063, p < 0.001].

Given the role of the IFJ in updating task rules^32, 33^, the elevated neural responses in recAN in this region during lose-shift raised the question whether condition-specific changes in IFJ activity functionally covaried with that in other brain regions and whether this change in connectivity differed between groups. A whole brain analysis [without family-wise error (FWE) correction] revealed evidence suggestive of an increase in coupling between IFJ and left AG (as well as caudate) during lose-shift in recAN, but a decrease in HC (Fig. 2; k ≥ 30voxels, p < 0.001, uncorrected). The left AG clusters overlapped anatomically with the cluster found for the contrast recAN>HC during lose-shift in our main general linear model (GLM). For detailed description of findings of the generalized psychophysiological interaction approach (gPPI) see SI 2.6.

## Discussion

The overarching goal of the current study was to investigate the neural correlates of reward-based decision making in recAN. Following up on our previous findings in acAN, we focused specifically on the neural correlations of changes in choice behaviour following monetary loss (lose-shift). Our analyses revealed the following main findings: First, overall task performance (lower hit ratio, fewer contingency reversals) and shifting behaviour, especially after negative feedback (lose-shift), was altered in recAN. Second, in contrast to our findings in acAN^19^, activation patterns in dACC were relatively unaltered. Third, activity in fronto-parietal brain regions, broadly implicated in cognitive control^34, 35^, was increased. Fourth, PPI analysis revealed suggestive evidence for increased coupling between IFJ (seed region) and AG. Together, these findings lend support to the notion that cognitive control is still altered in recAN and, as discussed below, is consistent with previous studies suggestive of altered neural efficiency in AN.

Regarding the first main finding, recAN earned a smaller total win and showed a decreased number of correct responses when compared to control participants. Furthermore, as in our previous investigation of acAN^19^, recAN showed increased lose-shift behaviour. This particular behaviour suggestive of elevated sensitivity to negative feedback may thus constitute an important trait characteristic in AN^36–39^.

In our previous study, elevated activation following negative feedback in acAN was found in the dACC^19^, a region involved in monitoring performance for the need for behavioural adaptation^40, 41^. In contrast, the current study in recAN did not reveal such a difference in the dACC relative to HC. Given the absence of undernutrition and associated (pseudo-) atrophic structural brain alterations (which we previously reported to be completely normalized in a recAN sample, which also included all participants of the current study^20, 42, 43^) and endocrine changes (see Table 1 for leptin levels) in recAN, aberrant dACC activity in acAN (as found in our previous study) therefore may reflect a state rather than a trait factor. Although these findings need to be interpreted with caution, it is interesting to note that taking general performance (as represented by overall hit ratio) into account revealed an interaction of dACC activation and task performance. RecAN participants that activated the dACC to a higher degree when confronted with monetary loss showed a better general task performance (as shown in Figure S5). Hence, the pattern of higher dACC activity accompanied by an increased performance is comparable to the pattern in acAN as found in the previous study. Therefore, we speculate that higher dACC activity, indicative of elevated performance monitoring^41, 44^, specifically during lose-shift behaviour, may enable better task performance.

More importantly, in the current study regions belonging to the fronto-parietal network (FPN)^34, 35^ including the IFJ and the AG were activated and functionally connected to a higher degree in recAN than in HC. The IFJ is broadly implicated in a range of executive operations including task-set updating, attentional control, and conflict processing^32, 33, 45–47^. In line with these functions of the IFJ, the current PRL task and especially the relevant lose-shift condition requires updating the values of the choice options and inhibiting the choice of the previously higher valued stimulus in order to maximize monetary gain. The IFJ is a plausible region in regard of our task as it was previously reported to be involved in cognitive functioning such as set-shifting, which was found to be impaired in recAN^10, 48–50^.

Regarding group differences during lose-shift behaviour uncovered in the AG, this region has been implicated in a range of functions including for example decision making during risk^51^ and reorientation^52^, i.e. guiding a person’s attention to salient information. Previous transcranial magnetic stimulation (TMS) studies stimulating AG described this region to be causally related to (actively) redirecting attentional orientation and suppressing stimulus-response conflicts^53, 54^. Additionally, abnormal patterns of resting state connectivity have been found in AN within the AG even after weight recovery, suggesting persistent abnormalities in the cognitive control network^14, 55^.

As outlined above, AN has been framed as a disorder of elevated cognitive control^56–58^ and even after recovery functional brain alterations, consistent with this hypothesis, have been found^17, 59^. In acAN, elevated brain activation in fronto-parietal control regions including the AG were found during behavioural shifts^7, 8^. However, to date no study has investigated lose-shift behaviour in recAN. Although our findings of increased activation of IFJ and AG in recAN participants may be seen as heightened cognitive control during lose-shift, behavioural performance was impaired in recAN. Hence, one possible interpretation of these findings may be neural inefficiency – i.e. elevated demand of cognitive resources to perform a difficult task. The hypothesis of neural efficiency was originally proposed in the field of intelligence stating that the brain’s efficiency depends on the focused use of task-relevant brain regions^60^. The combination of impaired task performance and increased activity within the FPN has been interpreted as neural inefficiency in a number of related psychiatric disorders such as schizophrenia^61–63^, OCD^64, 65^ or anxiety^66^. However, in the field of eating disorders some studies have found evidence suggestive of elevated neural efficiency in acAN. For example, during delay discounting acAN patients showed decreased FPN activation despite intact choice behaviour^6^. During a stop signal task, acAN have also been reported to show reduced task-relevant brain activation although behavioural performance was equal to HC^67^. In contrast, some studies in acAN and recAN found elevated neural responses in executive control regions during a go/no-go task^68^, delay discounting^69^, set-shifting^8, 70^, and a stop-signal task^71^, which could be interpreted as indicative of neural inefficiency.

Elevated activation of the cognitive control network during a PRL task can also be interpreted as indicative of altered reward-related decision making^72^. The group difference in lateral OFC, a region broadly implicated in reward and punishment processing^73, 74^, as found in this study supports this view. Recent theoretical considerations view AN as a disorder of reward processing^75^, for which some evidence exists including altered OFC structure^9, 76–78^ and functioning. For example, during reward learning and in response to food cues altered lateral OFC activation has been reported in acAN^79–81^. However, very few studies have investigated this in recAN. Therefore, future research in recAN targeting both disorder-relevant (e.g. food cues) and disorder-irrelevant reward stimuli is needed.

Our findings can also be interpreted from a neuro-biochemical perspective. Research on serotonin (5-HT) functions found evidence for a hyposerotonergic state in acAN (probably due to reduced tryptophan intake)^82^, but a hyperserotonergic state in recAN^83–85^. This is of interest since 5-HT has been implicated in aversive processing. In particular, low 5-HT levels are associated with reduced punishment sensitivity^86, 87^. Some behavioural characteristic typical for AN such as harm avoidance^1, 88^ were found to be related to increased 5-HT functioning^89–91^. Although speculative, our findings of elevated shifting after the receipt of punishment (monetary loss), which we found in recAN, but not in acAN, might be linked to increased 5-HT functioning. This hypothesis could be tested in future studies using tryptophan depletion^92^.

Some limitations of the current study need to be considered. Studying weight-recovered AN patients allows to minimize state effects related to acute undernutrition, but we cannot exclude scarring effects due to previous undernutrition or a selection bias (some patients never recover). Further, negative feedback was expressed by monetary loss. Therefore our results might not generalize to other feedback stimuli. Nonetheless, one strength of our study is the large, homogenous, and relatively young, medication-free recAN sample consisting of restrictive subtype AN only. Further, we controlled for satiety and chronobiological effects as our participants were all scanned in the morning after an overnight fast.

In conclusion, task performance and neural activation patterns during reversal learning in recAN compared to HC differ from that in our previous acutely ill AN sample suggesting alterations in lose-shift behaviour and neural correlates of lose-shift after recovery. A subgroup of recAN with better task performance seems to have a more similar pattern of dACC activation as found in acAN. Possibly, individuals who fully recover from the disorder also overcome exaggerated performance monitoring, which therefore might be seen as a state marker for acute AN. In fact, recAN showed elevated activation in the IFJ and AG, which may indicate attempts to strategically control task performance, but seems inefficient when considering the lower hit ratio and monetary win achieved by the patient group. Therefore, future studies should investigate longitudinal AN samples to differentiate between consequences and potential trait markers of the disorder. A study design that includes short- and long-term weight restoration could shed light on changes in PRL task performance to better understand cognitive control and its trajectories in AN.

## Methods

### Participants

The sample in the current study consisted of a total of 62 female volunteers: 31 recAN (15–28 years old) and 31 HC (15–27 years old). We conducted a case-control age-matching algorithm^93^ resulting in a maximum difference of 1.6 years between the individuals within one recAN-HC pair (mean age difference of 0.35 years). To be considered “recovered”, recAN subjects had to 1) have met AN criteria in the past (based on DSM-IV)^94^, 2) maintain a BMI > 18.5 kg/m² (if older than 18 years) or a BMI > 10^th^ age percentile (if younger than 18 years) for at least six months prior to the study, 3) menstruate, and 4) have not binged, purged, or engaged in significant restrictive eating patterns. All recAN participants of the current sample were of the restrictive AN subtype. To be included in the HC group, participants had to be of normal weight and eumenorrhoeic. RecAN and HC were recruited via advertisement among high school and university students or had previously participated in related clinical studies. This study was conducted between September 2011 and November 2013 and was carried out in concordance with the guidelines laid down in the Declaration of Helsinki and was approved by the ethical committee of the TU Dresden. All participants (and their guardians if underage) gave written informed consent.

Exclusion criteria and possible confounding variables, e.g. the use of psychotropic medications and medical comorbidities, were obtained using the expert version of the Structured interview for anorexia and bulimia nervosa for DSM-IV (SIAB-EX)^95^ and our own semi-structured interview. In the recAN group 22% of the participants had associated psychiatric comorbidity at the time of treatment (19% depressive disorders including dysthymia and 3% obsessive-compulsive disorder). For more details see Supplementary Information (SI) 1.1. Control analyses taking comorbid psychiatric symptoms into account can be found in SI 2.3.

HC participants did not have any history of psychiatric illness or a lifetime BMI below the 10^th^ age percentile (if younger than 18 years)/BMI below 18.5 kg/m^2^ (if older than 18 years) as assessed by the SIAB-EX and our semi-structured interview. Participants of both study groups had no lifetime history of any of the following clinical diagnoses: organic brain syndrome, schizophrenia, substance dependence, psychosis NOS, bipolar disorder, bulimia nervosa, or binge-eating disorder (or “regular” binge eating - defined as bingeing at least once weekly for 3 or more consecutive months). Further exclusion criteria for all participants were IQ lower than 85; psychotropic medication within 4 weeks prior to the study; obesity; current substance abuse; current inflammatory, neurologic, or metabolic illness; chronic medical or neurological illness that could affect appetite, eating behaviour, or body weight (e.g., diabetes); clinically relevant anaemia; pregnancy; breast feeding.

### Clinical Measures

For all participants, current and/or past diagnoses of eating disorders were ascertained using the expert form of the SIAB-EX^95^.

To complement the information obtained with the clinical interviews, eating disorder-specific psychopathology was assessed with the German version of the Eating Disorders Inventory (EDI-2)^96^. Here we focussed on the EDI-2 total score and the perfectionism subscale. Depressive symptoms were examined using the depression scale of the Symptom Checklist 90 Revised (SCL-90R)^97^.

Intelligence quotient (IQ) was estimated with a short version of the German adaptation of the Wechsler Adult Intelligence Scale (WIE)^98^ for participants aged ≥16 years or a short version of the German adaptation of the Wechsler Intelligence Scale for Children (HAWIK)^99^ for participants aged ≤15 years.

We used the BMI standard deviation score (BMI-SDS) instead of BMI for statistical analysis because the former provides an index of weight to height ratio that is corrected for age and gender^100, 101^.

Additionally, leptin levels were measured in plasma blood samples. For more details on endocrine, psychiatric, and psychological assessments see SI 1.1 and SI 1.2.

### Task description

We used a PRL task adapted from Hampton *et al*.^31^. This decision-making task includes probabilistic positive and negative monetary feedback and contingency changes according to a learning criterion.

**Figure 3:**
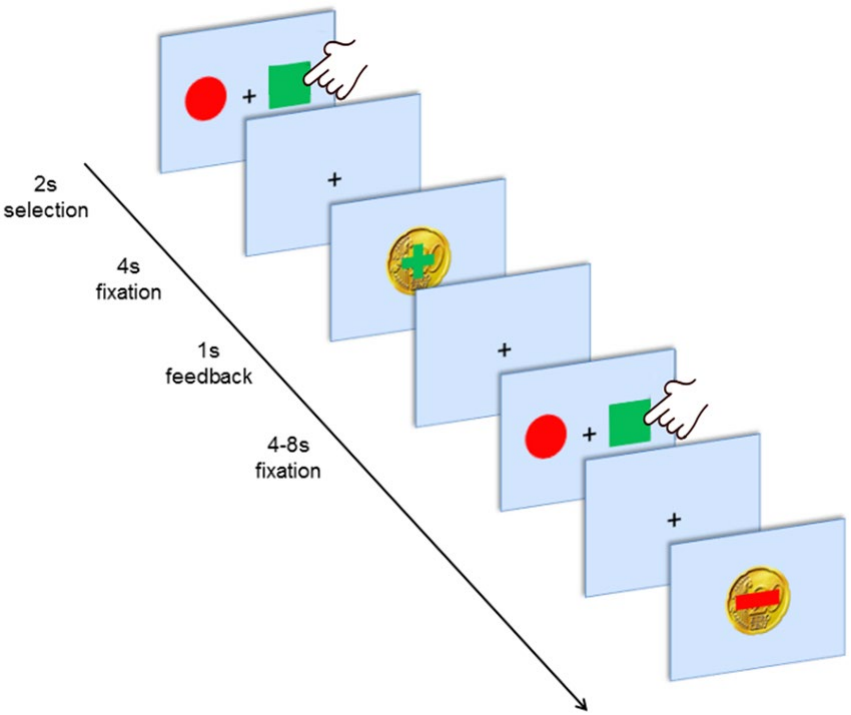
Experimental design. First, two abstract stimuli are presented for up to 2 s. After the participant selected one stimulus by left or right button press a fixation cross was presented for 4 s. Finally, positive or negative feedback (monetary reward or loss^109^) was displayed for 1 s followed by a jittered inter-trial interval (fixation cross) for 4 to 8 s.

The PRL task performed in the scanner consisted of 120 trials (total duration of ca. 26 minutes). In each trial, subjects were shown a coloured circle and a coloured square on the left and right side of a screen (spatial position randomized; Fig. 3). They were asked to choose one of the two symbols by pressing the left or right button within 2 seconds after stimulus presentation. In 80% the choice of the implicitly designated ‘correct’ or ‘incorrect’ symbol led to a positive feedback (+20cents) or a negative feedback (−20cents), respectively. In the remaining 20% of the cases probabilistic error occurred, i.e. choosing the ‘incorrect’ symbol led to monetary win and vice versa. A reversal of contingency (change of the ‘correct’ figure to the previously ‘wrong’ figure) occurred with a probability of 25% after at least four consecutive correct decisions, triggering a behavioural adaptation in the following trials (shifting behaviour). The total monetary win was paid at the end of the session. Before entering the scanner, participants absolved a training run to become acquainted with probabilistic errors (see also SI 1.3).

### Behavioural Data Analyses

As in our previous study^19^, the following behavioural performance measures were considered: occurrence of negative feedback that incurred a change in behaviour (lose-shift), occurrence of behavioural shift after receiving positive feedback (win-shift), hit ratio (number of correct responses), number of contingency reversals, total accumulated monetary reward (total win a participant achieved at the end of the experiment) and persistence, which is the average number of reversal errors after a contingency switch until the subsequent behavioural shift occurs (where a reversal error is defined as an incorrect response in consequence of applying the previously learned correct response). If not indicated otherwise, all values are presented as mean ± standard deviation. Further information and details on quality control on behavioural data is described in SI 1.4.

### MRI Data acquisition

Images were acquired between 8 and 9 a.m. in the morning after an overnight fast using standard sequences with a 3 T whole-body MRI scanner (TRIO; Siemens, Erlangen, Germany) equipped with a standard head coil.

The T1-weighted structural brain scans were acquired with rapid acquisition gradient echo (MP-RAGE) sequence with the following parameters: number of slices = 176; repetition time = 1900 ms; echo time = 2.26 ms; flip angle (FA) of 9°; slice thickness of 1 mm; voxel size of 1 × 1 × 1 mm³; field-of-view (FoV) of 256 × 224 mm²; bandwidth of 200 Hz/pixel.

The functional images were acquired by using a gradient-echo T2*-weighted echo planar imaging (EPI) with the following parameters: tilted 30° towards AC–PC line (to reduce signal dropout in orbitofrontal regions); number of volumes = 656; number of slices = 42; repetition time = 2410 ms; echo time = 25 ms; flip angle (FA) of 80°; 3 mm in-plane resolution; slice thickness of 2 mm (1 mm gap resulting in a voxel size of 3 × 3 × 2 mm³); field-of-view (FoV) of 192 × 192 mm²; bandwidth of 2112 Hz/pixel.

### MRI Data Preprocessing

Functional and structural images were processed using SPM8 toolbox (http://www.fil.ion.ucl.ac.uk/spm/) within the Nipype framework^102^. A DARTEL template was created using structural images from all subjects^103^.

The functional images were corrected for temporal slice-timing and motion simultaneously using realign4D^104^. This was followed by a coregistration to the subject’s structural brain and a normalization to MNI space using the DARTEL template and corresponding flow field. The resulting data were smoothed with an isotropic 8 mm FWHM Gaussian kernel.

The structural images were segmented into partial volume maps of cerebral spinal fluid (CSF), white matter (WM), and grey matter (GM). Erosion (kernel of 1 × 1 × 1 mm) was applied to the binarised CSF and WM maps. Afterwards both masks were merged to an anatomical noise mask defining brain regions that are unlikely to be modulated by neural activity as described by Behzadi *et al*. (2007; aCompCor)^105^. For more information see SI 1.5.

### MRI Analysis

On the first level for every participant a GLM was fitted to model the brain activation during three feedback conditions: (i) win (which includes win-shift since these events were too rare to be modelled), (ii) lose-stay, and our main condition of interest (iii) lose-shift. Additional regressors included six noise components (aCompCor) as well as one regressor for each motion or intensity outlier volume.

On the second level a linear mixed model including a three-level within-subject variable (feedback: win, lose-stay, lose-shift) and a binary between subject variable (group: recAN, HC) was estimated using GLM_flex. Based on the hypothesis of altered neural activity in recAN during lose-shift behaviour and our previous study in acute AN^19^ the primary contrast of interest was lose-shift. We examined activation on the whole brain level using conservative FWE correction (α < 0.01) and an additional cluster extent of k > 50voxels.

As additional analysis, we used gPPI^106^ to assess whether task-specific changes of functional connectivity between the effect of feedback (psychological factors: lose-shift) and the activity of the seed region (physiological factor) were present. Based on the findings in our main analysis (see results section), the seed was defined by a spherical region in the IFJ (centred at peak MNI coordinates *x* = −37, *y* = *10*, *z* = 28, radius 10 mm). For more detailed information see SI 1.6.

To specify group differences revealed by the whole-brain second level analysis and to further explore our previous findings of elevated dACC activation in acAN^19^, we extracted mean β estimates for each participant from all voxels belonging to relevant clusters using MarsBaR toolbox for SPM (http://marsbar.sourceforge.net/)^107^. The resulting values were then submitted to analysis of covariance and correlational analysis with clinical and behavioural data (SPSS)^108^. Correlation analysis focussed on overall eating disorder symptoms (EDI-2 total score) and perfectionism (EDI-2 subscale perfectionism).

## Electronic supplementary material

Supplementary Information
