# Supplementary material for: Neural correlates of altered feedback learning in women recovered from anorexia nervosa
Source: Sci Rep. 2017 Jul 14;7:5421. doi: 10.1038/s41598-017-04761-y (PMC5511172; doi:10.1038/s41598-017-04761-y)
Supplement: Supplementary file 1 — Supplementary Information [file 41598_2017_4761_MOESM1_ESM.pdf]

Supplementary Information

Neural correlates of altered feedback learning in women recovered from anorexia nervosa

Franziska Ritschel[1,2], Daniel Geisler[1,2], Joseph A. King[1,2], Fabio Bernardoni[1,2], Maria Seidel[1,2], Ilka Boehm[1,2], Richard Vettermann[1,2], Ronald Biemann[3], Veit Roessner[2], Michael N. Smolka[4], Stefan Ehrlich[1,2]

[1]Division of Psychological and Social Medicine and Developmental Neuroscience, Faculty of Medicine, Technische Universität Dresden, Dresden, Germany

[2]Eating Disorder Treatment and Research Center, Department of Child and Adolescent Psychiatry, Faculty of Medicine, Technische Universität Dresden, Dresden, Germany

[3]Institute of Clinical Chemistry and Pathobiochemistry, Otto-von-Guericke University, Magdeburg, Germany

[4]Department of Psychiatry and Neuroimaging, Technische Universität Dresden, Dresden, Germany

1. Methods

1.1 Psychiatric and psychological assessments

Diagnoses was established as mentioned previously with the SIAB-EX (Structured interview for anorexia and bulimia nervosa for DSM-IV) [1]. The SIAB-EX is a well-validated 87-item semi-standardized interview that assesses the prevalence and severity of specific eating-related psychopathology over the past three months. The interview provides diagnoses according to the ICD-10 and DSM-IV and is suitable for adolescents as well as adults. It has been used widely in eating disorder research [2–4]. A good inter-rater reliability (k=.81) for the diagnostic interview has been demonstrated [1]. Interviews were conducted by clinically experienced and trained research assistants under the supervision of the attending child and adolescent psychiatrist to confirm absence of eating disorder symptoms, i.e. the recovery status before scanning.

For recAN associated psychiatric comorbidity at the time of treatment was derived from medical records and confirmed by an expert clinician with over 10 years of experience after careful chart review (including consideration of medical and psychiatric history, physical examination, routine blood tests, urine analysis, and a range of psychiatric screening instruments). All diagnostic information was ascertained at the time of treatment.

Psychiatric conditions in potential HC were ascertained using the same instruments as in recovered patients. All interviews were carried out by trained graduate students (psychology or medicine). If there were any indications of psychiatric symptoms each case was discussed with a fully board-certified expert clinician and assessments were extended if necessary.

We used a short version of the German adaptation of the Wechsler Intelligence Scale for Children [56,7], which included the following subtests: vocabulary, letter number sequencing, matrix reasoning, and symbol search. The short version of the German adaptation of the Wechsler Adult Intelligence Scale [8,9] included the subtests: picture completion, digit symbol coding, similarities and arithmetics.

Handedness was assessed using a short version of the Annett Scale of Hand Preference [10] as previously implemented in Gollub et al. [11] This questionnaire asks for handedness in typical daily life situations as writing or brushing teeth. Response categories range from 0 'right hand', 1 'both hands' to 2 'left hand'. A mean score for handedness was calculated.

Study data were collected and managed using secure, web-based electronic data capture tools REDCap (Research Electronic Data Capture) [12].

1.2 Procedure for hormone measurements

To measure leptin levels, venous blood was collected into vacutainer tubes between 7 and 9 a.m. after overnight fasting. Plasma samples were centrifuged (800 × *g* for 15 min), aliquoted, and stored at -80°C until analysis. Hormone concentration in all participants was measured in one session at the same lab using a commercially available sandwich Enzyme Linked-Immunosorbent Assay (ELISA; BioVendor) following manufacturer instructions.

1.3 Experimental Paradigm

To prevent confusion, instructions were displayed again (after training) right before the scanner task started and participants were informed that the paradigm had been changed in a subtle way. Participants were instructed to maximize their gain and were informed that they will receive their total monetary win in addition to a fixed monetary compensation immediately after completing the experiments.

In the scanner, all stimuli were presented via a head-coil-mounted display system based on LCD technology (NordicNeuroLab AS, Bergen, Norway). Participants responded with two LUMItouch keypads (Lightwave Medical Industries, Canada). Stimuli were presented using Presentation® software (v11.1) [13].

1.4 Behavioural data analysis and quality control

Histograms, box plots, normal probability plots and Levene's tests were employed to verify the underlying statistical assumptions. Behavioural measures were compared using independent two-sample t-tests. Correlations were calculated using Pearson correlation coefficients. All tests were performed with SPSS statistical software version 21.0 [14].

To ensure data quality, we verified that no participant had a hit ratio (number of decisions for the 'wrong' figure divided by total number of trials) below or equal to 50% (performance below chance). Due to that two participants were excluded (1 recAN, 1 HC).

1.5 MRI Data Preprocessing & Quality Control

We evaluated the quality of the fMRI data by manual inspection and using artefact detection tools (ART) [15]. Volumes that exceed an brightness intensity threshold of three standard deviations or a threshold of 2 mm normalized movement in any direction were classified as outliers (motion-outlier: recAN: 2.6 ± 6.5 HC: 1.5 ±5.2; intensity-outlier: recAN: 8.2 ± 4.0 HC: 7.8 ±6.4). The two groups did not differ regarding numbers of motion- and intensity-outliers [motion-outlier: $t(60)= -.775$; $p= .442$; intensity-outlier: $t(60)= -.331$; $p= .742$].

In accordance to the aCompCor method we captured the time series from all voxels within the anatomical noise mask and extracted the first six principal components (explaining most of the variance). The six noise components were then subjected to our first level GLM model as nuisance regressors.

1.6 MRI Data Analysis

GLM_flex is a matlab script that implements a standard partitioned error GLM that is different from the corrected pooled approach used in SPM8. Thus, GLM_flex is preferable for multifactorial repeated measures and testing of interactions since the standard partitioned error approach makes fewer assumptions with regard to equality of variance, independence, and the stability of covariance patterns across voxels.

On the first level, the physiological activity was obtained by calculating the first eigen-variate across the voxel within the seed region and adjusting for the effects of interest. For every participant a whole-brain GLM analysis was performed using the following regressors: the deconvolved physiological activity of the seed region, the three psychological factors (win, lose-stay, lose-shift), and the three products of the physiological activity and each psychological factor (referred as "PPI regressors"). On the second level, the estimates of the three PPI regressors were subjected to a whole brain group analysis using GLM_flex (see above). We conducted an F-test to explore the interaction of group and the PPI regressors. Due to the exploratory purpose of the PPI connectivity analysis we report results with a voxelwise threshold of $p<0.001$ and a cluster extent of $k>=30$.

2. Results

2.1. Behavioural data

Table S1. Additional task performance measures. RecAN and HC compared by independent paired T-test (p<.05 describes significant different means). Displayed are means ± standard deviations.

	recAN	HC	T	p
lose-shift ratio	.588 ± .14	.525 ± .14	-1.724	.090
Win-shift ratio	.083 ± .09	.043 ± .05	-2.066	.044
Reaction time after lose	683.11 ± 133.4	639.03 ± 92.3	-1.513	.136
Reaction time after win	649.61 ± 129.4	619.42 ± 90.8	-1.063	.292

Abbreviations: recAN= recovered anorexia nervosa patients; HC= healthy controls.

2.2. Lose-shift main effect

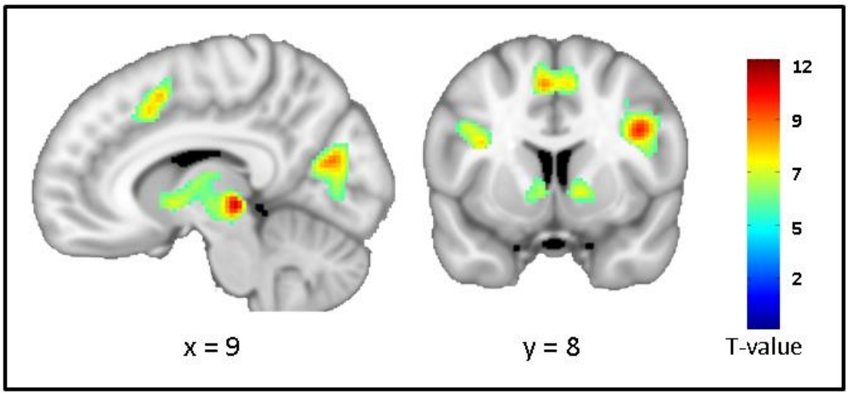

Figure S1. Main effect of lose-shift condition. Significant clusters found in anterior cingulate cortex, insula, dorsolateral prefrontal cortex, ventral striatum and others (confirming [16]). FWE; α=.01; k=50voxels.

2.3. Control analysis: effect of non-eating disorder symptoms

To investigate whether our main findings were influenced by psychiatric comorbidities, we reran the general linear model a) with the depression score (as assessed via SCL90-R) as a covariate and b) after excluding the recAN participant who had a comorbid diagnosis of OCD during the time of treatment. The results matched the findings obtained from the original sample. The findings are presented in Figure S2 (controlling for depression) and Figure S3 (controlling for OCD) below.

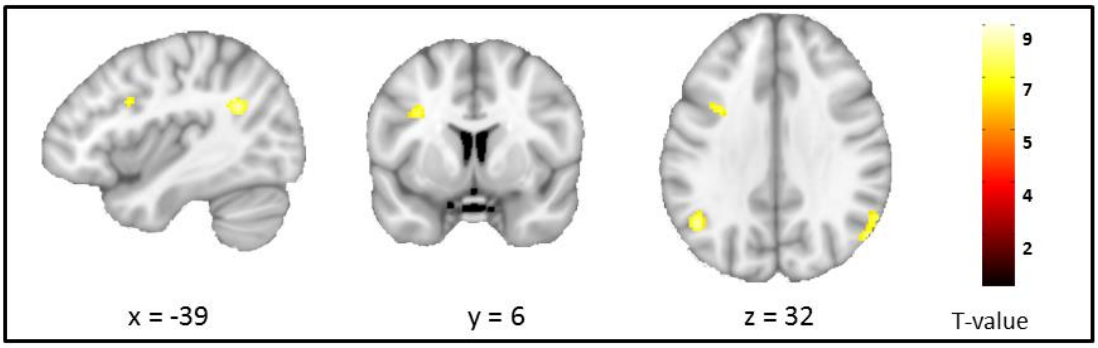

Figure S2. Results of follow-up analysis (lose-shift, recAN>HC) controlling for depression (subscale depression of the SCL90-R as a covariate).

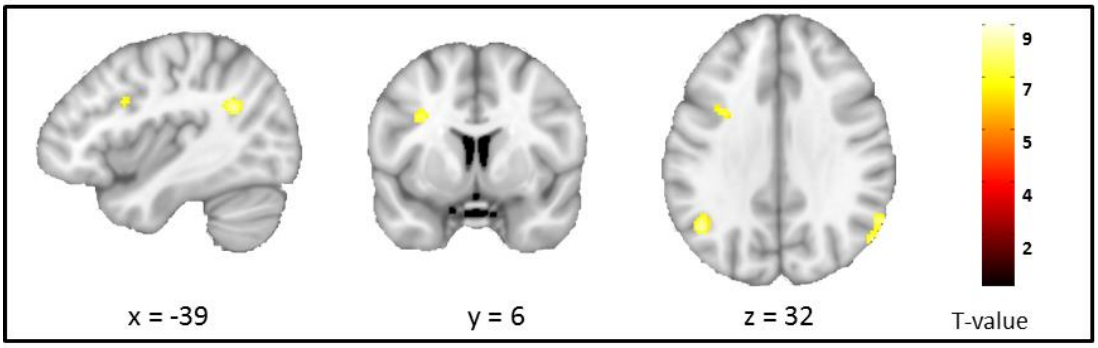

Figure S3 Results of follow-up analysis (lose-shift, recAN>HC) controlling for OCD (exclusion of all participants with OCD diagnosis at the time of treatment (n=1)).

2.4. Lose-shift HC>recAN

For the opposite contrast of lose-shift (HC>recAN) a significant activation was found in the following regions: left superior occipital gyrus, right middle occipital gyrus, left calcarine, left superior parietal gyrus and right lingual gyrus.

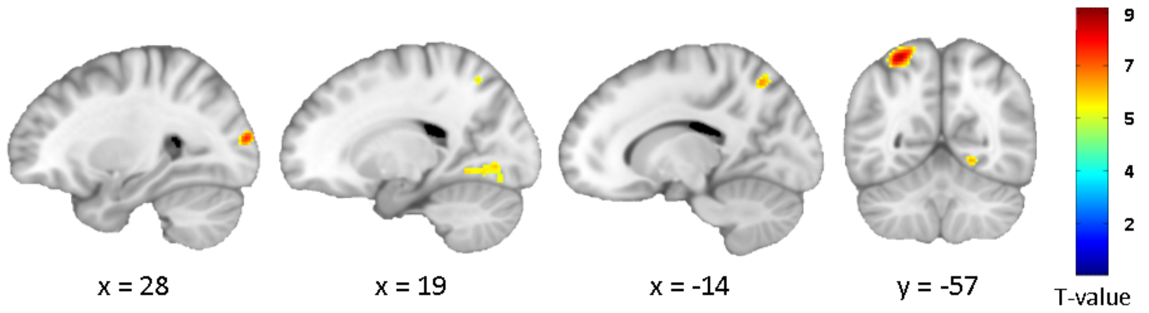

region	hemisphere	Peak voxel MNI coordinates			clustersize	T
		x	y	z		
Posterior parietal lobe	L	-23	-60	64	371	9.0744
Superior occipital lobe	L	-25	-86	28	52	6.1958
Calcarine cortex	R	21	-54	-8	133	6.4248
Lingual Gyrus	Bilateral	1	-82	8	137	6.3968
Middle occipital gyrus	R	29	-92	14	62	7.5128

Figure S4. **Group differences (HC > recAN) for lose-shift behaviour.** Brain maps showing regions that are activated during lose-shift in HC more than in recAN (FWE; α=.01; k=50voxels). Below local peaks of clusters are listed that are more activated by lose-shift behaviour in HC.

Abbreviations: lose-shift= negative feedback incurring a change in behaviour; L= left; R= right; HC= healthy control; recAN= recovered anorexia nervosa patient

2.5. dACC activity

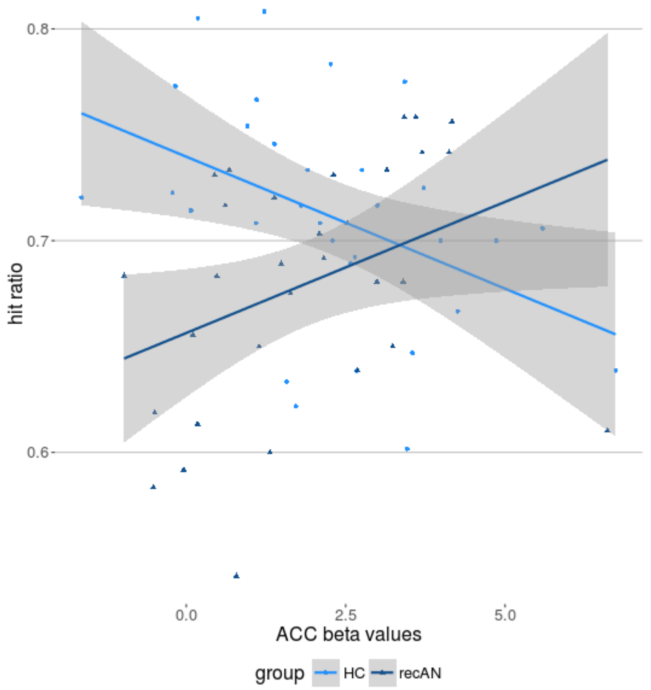

Figure S5. Relationships between the dACC activity and impaired behavioural performance (hit ratio). Significant group × hit ratio interaction [F(2,59)= 10.91, p= .002]: high hit ratio was associated with higher dACC response in recAN, while HC showed the opposite pattern

2.6. gPPI

Table S2 Clusters showing group differences (recAN>HC) in change of functional connectivity with left IFJ (seed region) during lose-shift. Peak voxels of clusters surviving FWE-correction (p<.001; k≥10voxels).

Target region	hemisphere	Peak voxel MNI coordinates			cluster size	T
		x	y	Z		
Angular Gyrus	L	-45	-48	28	44	4.4063
Caudate	R	15	-2	28	40	3.7861

Abbreviations: lose-shift= negative feedback incurring a change in behaviour; L= left; R= right; HC= healthy control; recAN= recovered anorectic patient

References

1. Fichter, M. & Quadflieg, N. The structured interview for anorexic and bulimic disorders for DSM-IV and ICD-10 (SIAB-EX): reliability and validity. *Eur. Psychiatry J. Assoc. Eur. Psychiatr.* **16,** 38–48 (2001).
2. Giel, K. E. *et al.* Attentional Processing of Food Pictures in Individuals with Anorexia Nervosa—An Eye-Tracking Study. *Biol. Psychiatry* **69,** 661–667 (2011).
3. Kaye, W. H., Bulik, C. M., Thornton, L., Barbarich, N. & Masters, K. Comorbidity of Anxiety Disorders With Anorexia and Bulimia Nervosa. *Am. J. Psychiatry* **161,** 2215–2221 (2004).
4. Zipfel, S. *et al.* Focal psychodynamic therapy, cognitive behaviour therapy, and optimised treatment as usual in outpatients with anorexia nervosa (ANTOP study): randomised controlled trial. *Lancet Lond. Engl.* **383,** 127–137 (2014).
5. Petermann, F. & Daseking, M. *Fallbuch HAWIK-IV.* (Hogrefe, 2009).
6. Petermann, F. & Petermann, U. *Hamburg Wechsler Intelligenztest fuer Kinder IV (HAWIK-IV).* (Huber, 2008).
7. Waldmann, H.-C. Kurzformen des HAWIK-IV: Statistische Bewertung in verschiedenen Anwendungsszenarien. *Diagnostica* **54,** 202–210 (2008).
8. von Aster, M., Neubauer, A. C. & Horn, R. *WIE. Wechsler Intelligenztest für Erwachsene. Deutschsprachige Bearbeitung und Adaptation des WAIS–III von David Wechsler*. (Harcourt Test Services, 2006).
9. Donnell, A. J., Pliskin, N., Holdnack, J., Axelrod, B. & Randolph, C. Rapidly-administered short forms of the Wechsler Adult Intelligence Scale-3rd edition. *Arch. Clin. Neuropsychol. Off. J. Natl. Acad. Neuropsychol.* **22,** 917–924 (2007).
10. Annett, M. A Classification of Hand Preference by Association Analysis. *Br. J. Psychol.* **61,** 303–321 (1970).
11. Gollub, R. L. *et al.* The MCIC collection: a shared repository of multi-modal, multi-site brain image data from a clinical investigation of schizophrenia. *Neuroinformatics* **11,** 367–388 (2013).

12. Harris, P. A. *et al.* Research electronic data capture (REDCap) - A metadata-driven methodology and workflow process for providing translational research informatics support. **42(2),** 377–81 (2009).
13. Neurobehavioral Systems. *Presentation, Version 11.1*. (Neurobehavioral Systems, 2007).
14. IBM Corp. *IBM SPSS Statistics for Windows, Version 21.0*. (IBM Corp., 2012).
15. Whitfield-Gabrieli, S. *Artifact Detection Tool*. (2009).
16. Hampton, A. N., Bossaerts, P. & O'Doherty, J. P. The role of the ventromedial prefrontal cortex in abstract state-based inference during decision making in humans. *J. Neurosci. Off. J. Soc. Neurosci.* **26,** 8360–8367 (2006).
